# Evaluating a 24-h mobile reporting system for malaria notifications in comparison with a paper-based system in South Africa, 2015

**DOI:** 10.1186/s12936-018-2451-x

**Published:** 2018-08-23

**Authors:** Ramokone Ednah Baloyi, Mbavhalelo Bridget Shandukani, Rebecca Graffy, Eunice Misiani, Natalie Mayet, Eric Mabunda, Aaron Mabuza, Bheki Qwabe, Bongani Ngwenyama, Carl Reddy, Devanand Moonasar

**Affiliations:** 1 South African Field Epidemiology Training Program, 1 Modderfontein Road, Sandringham, Johannesburg, South Africa; 2 National Institute for Communicable Diseases, 1 Modderfontein Road, Sandringham, Johannesburg, South Africa; 3 National Department of Health, Civitas Building, 222 Thabo Sehume Street, Pretoria, Gauteng 0001 South Africa; 4 Clinton Health Access Initiative, 31166 Francis Baard, Block b, 1st Floor, Hatfield, Pretoria, Gauteng 0001 South Africa; 5 Limpopo Government Department of Health, 618 College Street, Polokwane, Limpopo 0700 South Africa; 6 Mpumalanga Government Department of Health, Building No.7, Government Boulevard, Riverside Park, Extension 2, Nelspruit, Mpumalanga 1200 South Africa; 7 KwaZulu-Natal Government Department of Health, Natalia Langalibalele (Longmarket) Street, Pietermaritzburg, KwaZulu-Natal 3201 South Africa

**Keywords:** Disease surveillance strengthening, Malaria elimination, Feasibility of 24-h mobile reporting, Timeliness of data input

## Abstract

**Background:**

As South Africa strives to achieve malaria elimination by 2018 (zero local cases) the country needs to strengthen its disease surveillance system by reducing the timeliness from case diagnosis to notification of key stakeholders in the malaria programme. This study evaluated the feasibility of a 24-h mobile reporting system, designed for speeding up malaria notifications, from primary healthcare facilities to district, provincial, and national malaria programmes in South Africa.

**Methods:**

A prospective descriptive study utilizing primary data collected from structured interviews with healthcare workers in public healthcare facilities was used to compare two reporting systems (24-h mobile reporting system and the paper-based reporting system) in malaria endemic provinces (Limpopo, Mpumalanga and KwaZulu-Natal). Data on completeness of reporting, simplicity, user acceptability and technical limitations were analysed. A Wilcoxon signed-rank test was used to compare the time difference between the two reporting systems.

**Results:**

There were 1819 cases of malaria reported through the paper-based system, and 63.2% (1149) of those cases were also reported through the 24-h mobile reporting system. Out of the 272 healthcare workers who were interviewed, 40% (108) had seen malaria patients and reported a case through the 24-h mobile reporting system. The median time for cases to be reported through the 24-h mobile reporting system was significantly shorter at < 1 day (range < 1 to 31 days) compared to the paper-based system at 3 days (range 2 to > 39 days) (p < 0.001). It was found that 26% (28) were able to use the system and send reports within 2 min, 94% (256) were willing to continue to use the system. Of the 108 healthcare workers who reported a case, 18.5% (20) experienced network challenges.

**Conclusions:**

The 24-h mobile reporting system is user friendly and trained healthcare workers are willing to use the system, despite network limitations. The 24-h mobile reporting system reduces the time required for diagnosed cases to be notified by the health care facility to district, provincial and national levels. The 24-h mobile reporting system is a feasible option for malaria notification in South Africa and will assist with early detection of malaria outbreaks.

## Background

South Africa’s malaria control programme has been successful in reducing the incidence of malaria in the country from a control to a pre-elimination phase [1]. Transitioning the programme from a pre-elimination to an elimination phase (0 cases/1000 population at risk) will require among others strengthening of the surveillance system, ensuring that cases are reported within 24 h of diagnosis, and optimizing data collection and reporting through the use of new technological methods [1–3].

**Fig. 1:**
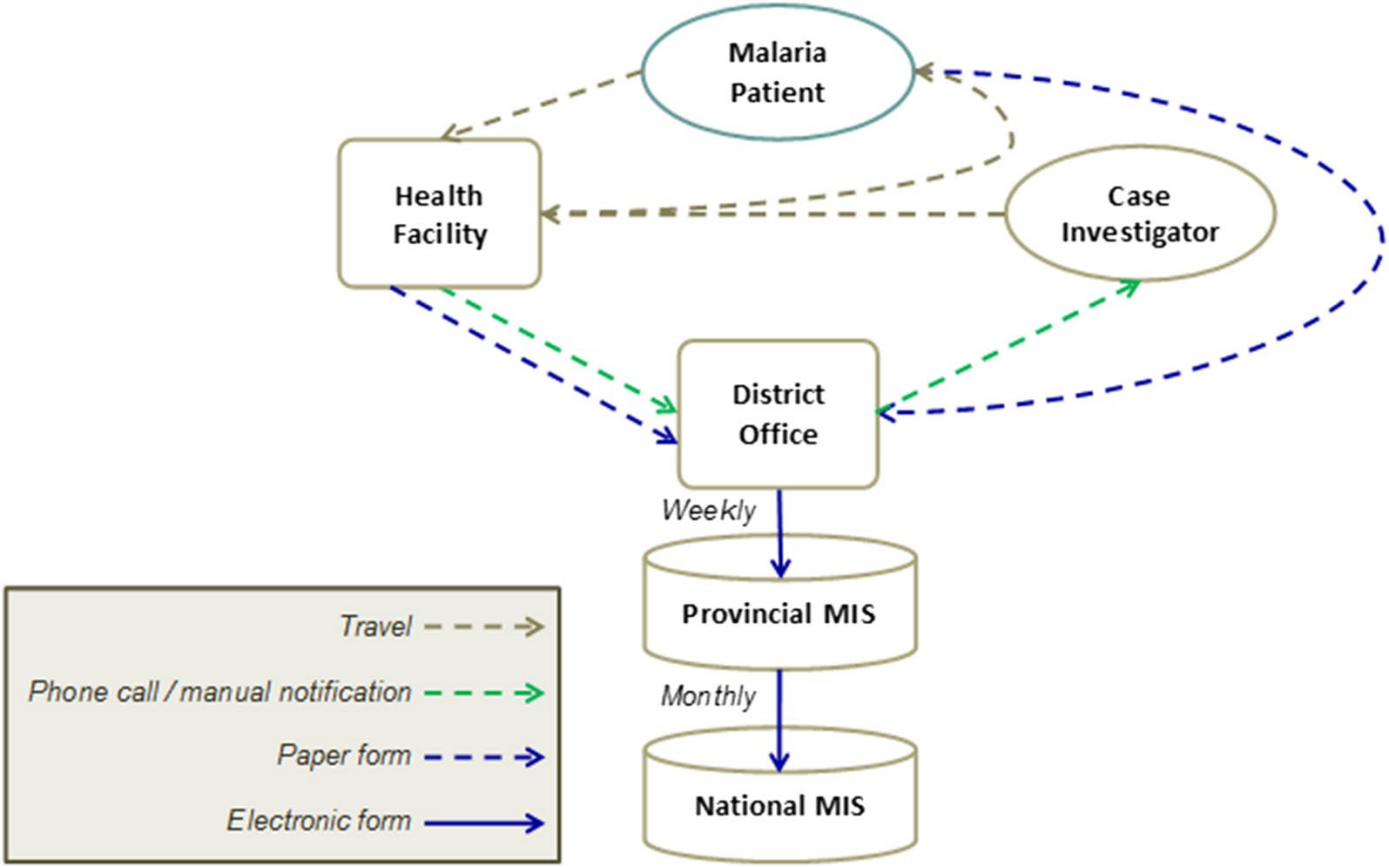
Flow of information across the current paper-based surveillance system across all the malaria endemic districts, South Africa 2016 [3]. 24-h mobile reporting system

According to the World Health Organization (WHO), case reporting practices are a key indicator in strengthening a surveillance system [1, 2, 4]. Current case reporting practices in South Africa vary by province, district, and facility; however, in all instances the process is manual [1, 5]. When a malaria case presents at a health care facility, health care workers are required by protocol to complete the requisite notification paperwork, and in many instances to alert a central contact by phone [3]. The notification paperwork consists of 5 sections; healthcare facility information, patient’s demographic information, medical conditions details, travel history section and a specimen details section [3]. A malaria case notification, which is approximated to take 2–5 min to complete triggers a case investigation, in which a case investigator travels to the patients’ residence to complete an EP6 (case investigation) form, often after first travelling to the facility to collect the paper-based notification form [3]. The paper-based notification form and the EP6 form are submitted to the central office, and data is later entered as an electronic record by data capturers (Fig. 1) [3].

**Fig. 2:**
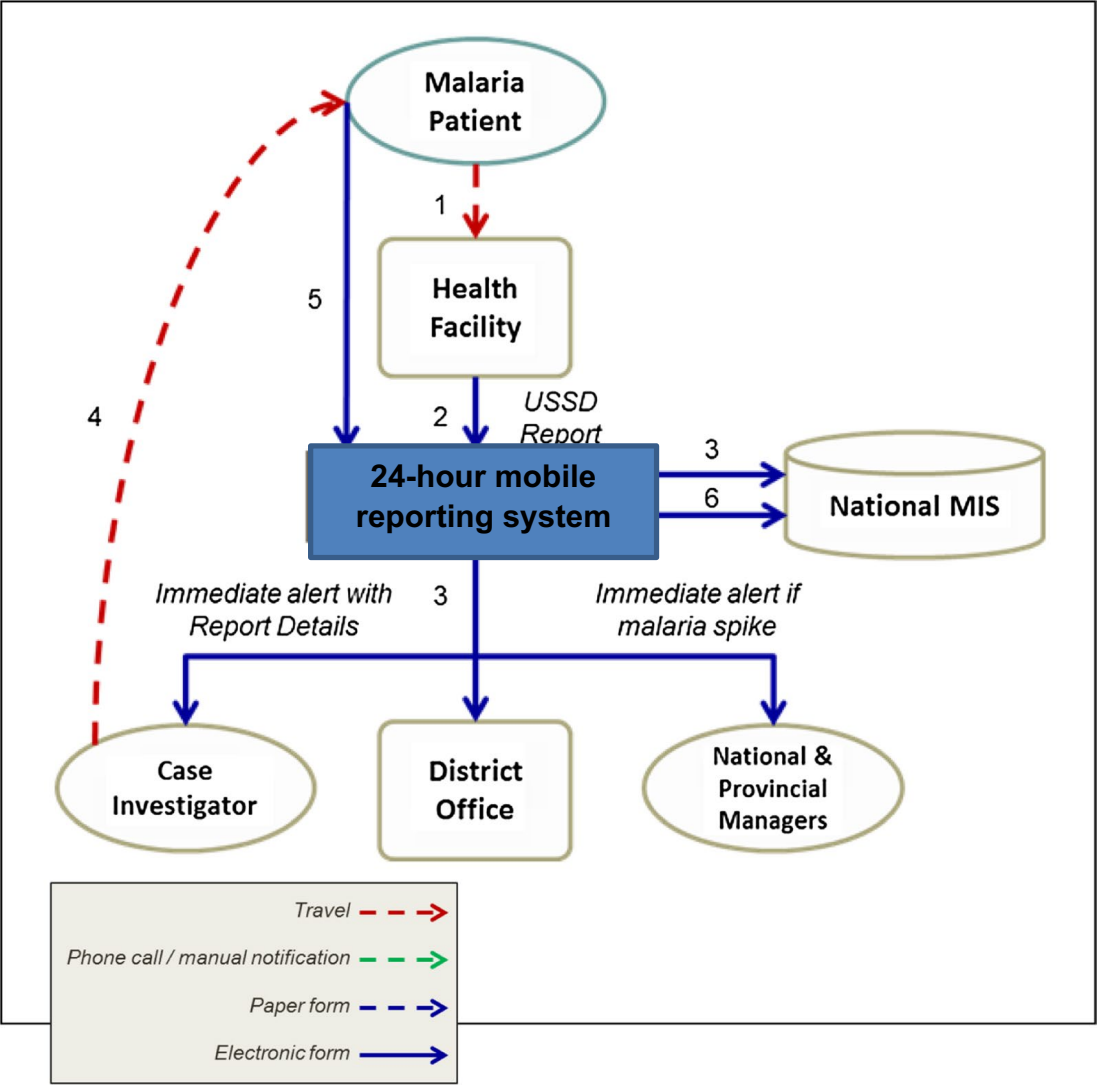
Flow of information across the anticipated 24-h mobile reporting surveillance system across all malaria endemic districts, South Africa, 2016 [3]

To prevent delays in reporting, the National Department of Health (NDOH) has developed a technological solution for immediate reporting of malaria cases [3]. The 24-h mobile reporting system was first introduced in 2012 and was implemented in 2015. The 24-h mobile reporting system is an electronic reporting system that uses Unstructured Supplementary Service Data (USSD) technology [3]. The electronic reporting system compromises of 10 questions about the reporting facility details, patient’s demographic information, medical conditions details and travel history [3]. The average time it should take for a case to be reported through this platform is 2 min [3]. The 24-h mobile reporting system enables health care workers to submit an electronic report directly to the national malaria information system (MIS), housed at the National Institute for Communicable Diseases (NICD), with alerts generated to the relevant individuals for follow up (Fig. 2) [3].

Mobile technology is used globally as a surveillance tool [5, 6]. The benefits and shortfalls are well documented in the literature [6–8]. The use of mobile technology as a reporting tool is highly prevalent in developed countries and also in some developing countries as they are required by legislation to report all notifiable medical conditions (NMC) [3, 5]. There is a shortage of healthcare staff in most developing countries at facilities and a continuous reporting burden, which can be stressful [7, 9–12]. Mobile technology is an innovative way to minimize delays in programmatic action due to this burden [2–5].

## Methods

### Aims and objectives

The aim of the study was to evaluate the feasibility of implementing the 24-h mobile reporting system to decrease the time required for malaria cases to be reported from the health facility level to the district, provincial, and national level for investigation and response. Timeliness, completeness, data validity, and simplicity between the paper-based and the 24-h mobile reporting systems was compared. Acceptability and technical limitations for end users in the use for the new 24-h mobile reporting system was also assessed.

### Study design

This was a cross-sectional quantitative study, which evaluated and compared the current paper-based reporting system and the new 24-h mobile reporting system in the years 2015–2016. Interviewer-administered questionnaires were applied and secondary data analysis of the MIS was conducted.

### Study sites

**Fig. 3:**
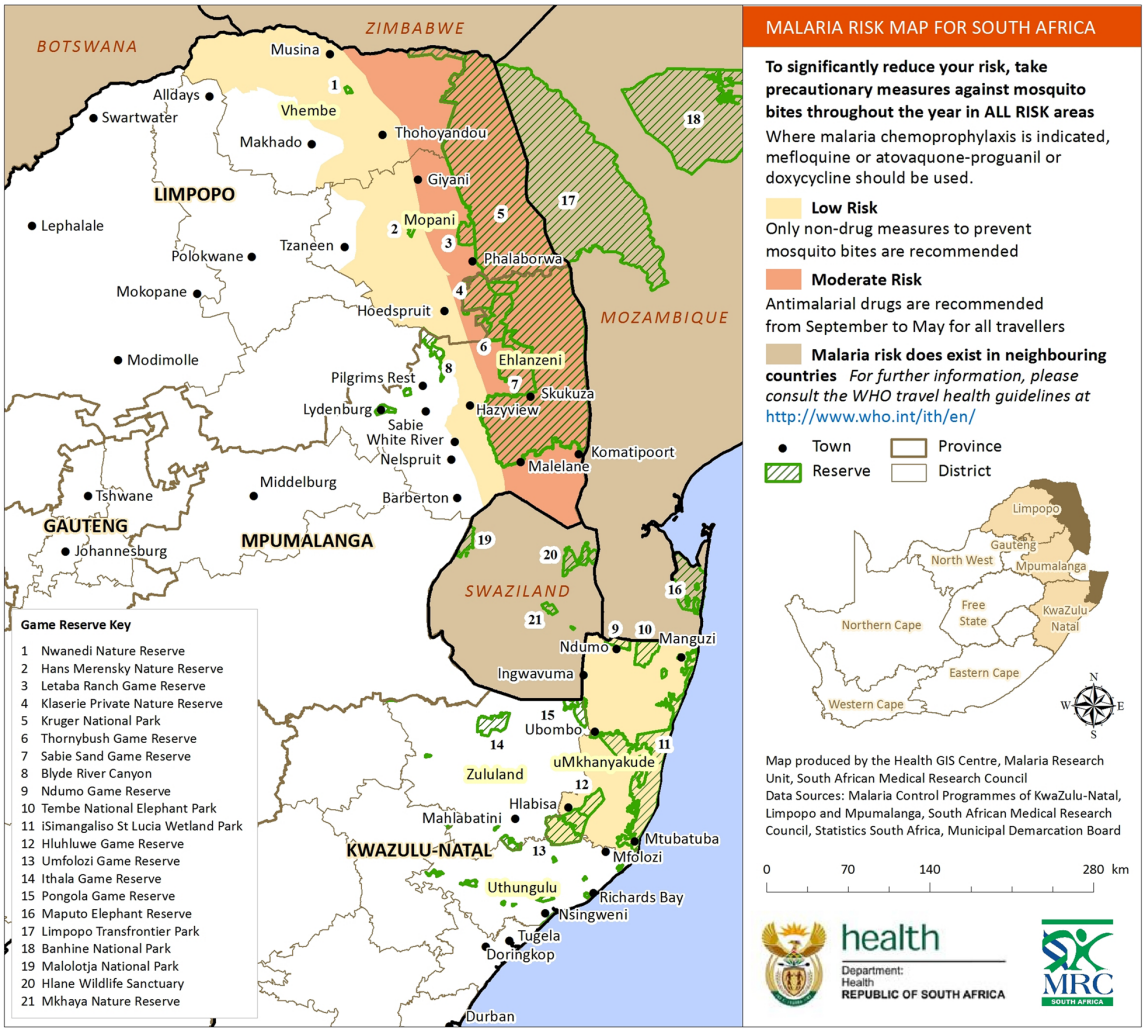
A map depicting malaria endemic provinces and districts in South Africa, 2015 [16]

The study was carried out in the three malaria endemic provinces of South Africa (Limpopo, Mpumalanga and KwaZulu-Natal). The focus was on the five malaria endemic districts that have the highest prevalence of malaria: Umkhanyakude and Uthungulu in KwaZulu-Natal, Ehlanzeni in Mpumalanga, and Mopani and Vhembe in Limpopo (Fig. 3).

### Inclusion/exclusion criteria

The malaria endemic districts that were included in the study were purposively selected [13]. The endemic districts of South Africa are reported to be either in their pre-elimination stage or elimination stage (no local transmitted malaria cases) [1]. A retrospective records review of the cases reported during 2012–2014 was conducted to assess for malaria transmission trends at the facilities level. The districts selected must have reported at least one malaria case in the past 3 years. Any health care facilities in these five districts must have recorded at least one malaria case in the 2012–2014 malaria seasons in order to focus resources where they are needed most. All the malaria cases that were reported in each of these facilities during the study period were included in the study.

Private health care facilities and specialized health care facilities were excluded from the study. Although private healthcare facilities do report malaria cases in the country, South Africa being a developing country, with less than 26% of the total population having medical aid, which gives them access to private sector care, the burden of malaria reported from private healthcare facilities is less than 1% [12]. The study had limited economical and human resource constrains, due to this gap private healthcare facilities were excluded and facilities that were most burdened were included in the study.

### Project implementation

#### Healthcare worker training

The investigators first trained the NDOH staff, which comprised of an Information specialist, the surveillance assistant director, and 5 analysts on both the paper-based system and the 24-h mobile reporting system. Healthcare workers were then trained by the NDOH team and the investigators, who visited facilities that met the inclusion criteria. The training comprised of the screening of patients who were suspected to have malaria, the correct use of the rapid diagnostic test, treatment of malaria positive cases and management of complicated cases [10]. A letter was sent out to the malaria team (Environmental Health Practitioners, case investigators, malaria surveillance agents) in the endemic areas to send to the managers at the healthcare facilities. The malaria teams were designated to provide support to the healthcare workers during implementation.

#### Data reporting procedures

Following the training of healthcare workers, weekly follow-up visits (approximately 20 visits) to the healthcare facilities were made. These follow up visits were to establish and resolve any technical limitations that might have hindered the successful implementation of the new 24-h mobile reporting systems and to address any concerns that health care workers could have with regards to using both reporting systems in parallel. Continuous training of healthcare workers both trained and those that were not trained are also conducted during the follow-up visits. Aggregated data on the cases reported at the facilities were also compiled and reported back to the healthcare facilities.

#### Data validation

Data validation of all cases that were reported were conducted. All the paper notification forms were collected from the healthcare facilities and it was paired with the data from the 24-h mobile reporting system, which was extracted from the database where it is stored. Discrepancies between the cases reported between the two systems were established by pairing the reported cases through the paper-based system with the cases that were reported through the 24-h mobile reporting system. The healthcare workers were contacted in order to establish reasons for cases that were not reported through both systems.

### Definitions of attributes of surveillance evaluations

#### Completeness

To assess for completeness, two attributes were measured, completeness of reporting rates between the papers based system and the 24-h mobile reporting system and data validity between the two systems. Completeness of reporting rates was defined as the number of cases that were reported through the paper-based system that was also reported through the 24-h mobile reporting system. Data validity was the percentage of fields that were correctly completed in the paper-based system as compared to the 24-h mobile reporting system.

#### Timeliness

Timeliness was defined as the difference in the time from case diagnosis to entry into the MIS.

#### Simplicity

Simplicity was defined as the time required for completing a case report, and the users’ assessment of ease of use.

#### Acceptability

Acceptability referred to the willingness of the health care workers to use the 24-h mobile reporting system.

#### Technical limitations

To assess whether there were any technical limitations for end users of the 24-h mobile reporting system, the attribute of stability was measured. Stability refers to the ability of the system to be operational when it is needed and its ability to collect, manage and provide data properly without failure.

### Sample size

This study was powered to detect a time difference in reporting of seven days between the two systems. An average of two cases per facility was sufficient to achieve the required minimum sample size of 600 malaria cases. The sample size was calculated using STATA version 13 with the following assumptions: 90% power, difference of seven days with a standard deviation of 25 days (based on 2014 MIS data), 5% level of significance, 10% adjustment for potential loss to follow-up, and adjustment for clustering within facilities and districts that assumes an average of 20 cases per facility and an intra-cluster correlation of 0.05.

### Data analysis

The completeness of reporting rates was evaluated by comparing the percentage of successfully reported cases between the two systems. If all the questions were correctly completed, then it was rated a 1. If the data had more than 1 incorrectly completed field it was given a 0, because all the fields to be completed are critical for case tracing. A Chi square test was used to compare completeness and data validity. A Wilcoxon signed-rank test was used to compare timeliness between the 24-h mobile reporting system and the paper-based system. Simplicity, acceptability and stability of the system were evaluated using an interviewer administered structured questionnaire.

## Results

### Response rates, completeness and validity of the data

There were a total of 298 healthcare facilities that were recruited into the study, of which 145 (49%) were from Limpopo, 95 (32%) from Mpumalanga and 58 (19%) from KwaZulu-Natal. Of the 298 healthcare facilities that were recruited into the study, 58% (n = 172) reported malaria cases through the 24-h mobile reporting system and 64% (n = 190) reported cases through the paper-based system (Table 1).

**Table 1:** The geographical distribution of health care facilities in malaria endemic provinces that were selected to use the new 24-h mobile reporting system, South Africa, 2015–2016

Reporting system	Number of healthcare workers
24-h mobile reporting system only	4
Both 24-h mobile reporting system and paper-based reporting system	104
Paper-based reporting system only	68
Never used neither of the systems (only trained)	96
Total	272

**Fig. 4:**
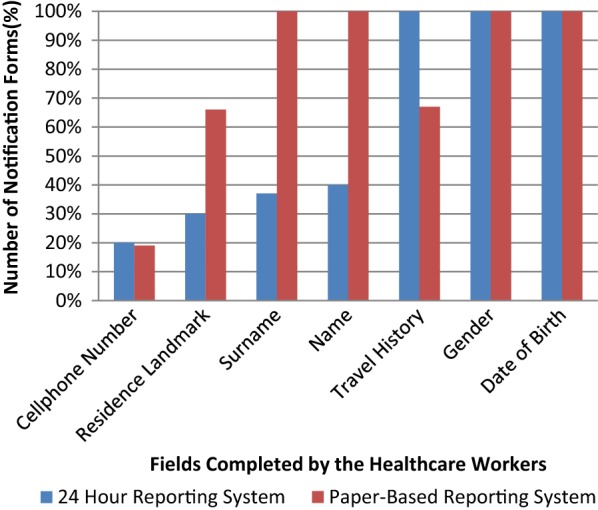
Completeness of fields of the cases reported through the 24-h mobile reporting system and the paper-based reporting system, South Africa, September 2015–March 2016

There were 1819 cases of malaria reported through the paper-based system, and 63.2% (1149) of those cases were also reported through the 24-h mobile reporting system (Fig. 4). The date of birth and gender were complete in 100% (1819) of the paper-based system notifications and 100% (1149) for the 24-h mobile reporting system. The travel history field was complete in 62% (1128) of the paper-based system notification forms, while it was 100% (1149) for the 24-h mobile reporting system.

The surname was correctly completed in 100% (1819) of the paper-based system and 38% (437) of the 24-h mobile reporting system, while the name was 100% (1819) for the paper-based system and 40% (460) for the 24-h mobile reporting. The cell phone number field was complete in 19% (345) of the paper-based system and 20% (364) in the 24-h mobile reporting system (Fig. 4).

### Timeliness between the 24-h mobile reporting system and the paper-based system

The median timeliness of the 24-h mobile reporting system was significantly shorter compared to the paper-based system (< 1 day [< 1 to 31 days] versus 3 days [3 to > 39 days], p < 0.001) (Table 2).

**Table 2:** The distribution of the reporting system the healthcare workers utilized, 2015–2016

Province	District	Number of healthcare facilities recruited	24-h mobile reporting only	24-h and paper-based system	Paper-based system only
Limpopo	Vhembe	86	1	79	6
	Mopani	59	2	46	11
	Sub-total	145	3	125	17
Mpumalanga	Enhlanzeni	95	0	90	5
	Sub-total	95	0	90	5
KwaZulu-Natal	Umkhanyakude	51	0	44	7
	Uthungulu	7	0	3	4
	Sub-total	58	0	47	11
	Total	298	3	262	33

**Fig. 5:**
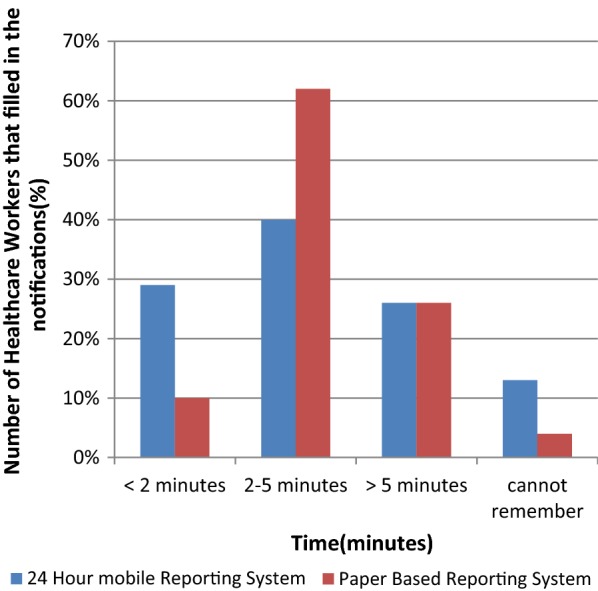
Simplicity for the healthcare workers to use the system assessed by the time taken for healthcare workers to complete the notification

There were a total of 272 healthcare workers that were interviewed across all five districts. All healthcare workers that were interviewed had been trained to use the new 24-h mobile reporting system. Of all healthcare workers that were interviewed, 40% (108) reported a case through the 24-h mobile reporting system, while 62% (168) reported a case through the paper-based system. Users reported that it took less time for case notifications to be completed using the 24-h mobile reporting system, compared to the paper-based system [26% versus 17% were filled in < 2 min (p = < 0.001)] (Fig. 5).

### Simplicity to use the 24-h mobile reporting system and the paper-based reporting system

Among the healthcare workers that used the 24-h mobile reporting system, 85% (92) did not find any parts of the 24-h mobile reporting system to be challenging, while out of the healthcare workers that used the paper-based system 37% (40/168) did not find the paper-based system to be challenging (p-value < 0.001).

### Acceptability to use the 24-h mobile reporting system

Of the 272 healthcare workers that were interviewed, 94.1% (256) were willing to use the 24-h mobile reporting system.

Of all the 108 healthcare workers that used the 24-h mobile reporting system, 7% (8) experienced the system not working due to failure to connect to the network and 19% (21) did experience downtimes.

## Discussion

The study assessed the completeness of reporting rates, data validity, timeliness, simplicity and user acceptability between two reporting systems and the user across five malaria endemic districts in South Africa. The study also assessed technical limitations that can hinder the successful implementation of the 24-h mobile reporting system. Overall the study findings demonstrated that the completeness of reporting and data validity was better for the paper-based system and that the 24-h mobile reporting system was a better tool to reduce the time from case diagnosis to notification to the malaria programme. The study also demonstrated the simplicity of the 24-h mobile reporting to be better than that of the paper-based system. Healthcare workers were willing to continue using the system. There are technical limitations that can hinder the success of cases being notified within 24-h by healthcare workers.

Reporting of NMC remains a challenge, even in developed countries where considerable resources are allocated to disease surveillance annually [14]. In developing countries, such as South Africa, lack of infrastructure, shortage of healthcare workers, and lack of training of healthcare workers at healthcare facilities are some of the reasons that reporting rates are poor.

Recent evidence from studies conducted in both developed and developing countries have shown that mobile technology is more efficient and reliable than using a paper-based system notification for notification of medical conditions, but the study findings oppose these findings [6, 8]. There were more cases reported through the paper-based system as compared to the 24-h mobile reporting system. The most commonly documented reason for this is the frequent healthcare worker turnover, which results in some healthcare workers not being trained on the new 24-h reporting system [8]. The paper-based system is the golden standard and healthcare workers are trained on it during their academic training. Majority of healthcare workers graduating in South African universities or colleges are already familiar with the paper-based system. In order for the 24-h reporting system to be effectively used, training would have needed to be provided to the healthcare workers. Healthcare workers are already familiar with reporting malaria cases using the paper-based system and, therefore, are more inclined to use a reporting system they are more familiar with.

To prevent this from hindering the feasibility of the 24-h mobile reporting system in decreasing the time required for cases to be reported from the healthcare facility, more re-fresher training visits should be scheduled by the provincial teams, in that way, healthcare facility workers that have not been trained on the 24-h mobile reporting system could be trained.

The data quality was poor for the 24-h mobile reporting system as compared to the paper-based system. This was due to the phrasing of the questions on the 24-h mobile reporting system. There were certain questions that had double meanings and examples that were not relevant in the South African context. Previous studies have also shown that data quality remains a big challenge when a new technology is introduced, especially among the non-youth age groups (> 35 years) [5]. It also takes time for healthcare workers to adapt to new reporting tools. Non-standardization of training techniques could have also led to different ways of understanding how to use the 24-h mobile reporting system, as a decentralized method of training was used.

The time for cases to be notified using the paper-based system was longer than the time for cases to be notified using the 24-h mobile reporting system; this was due to the USSD technology, which allows immediate case reporting from the healthcare facility to the NMIS. Technological solutions used in different diseases have strengthened disease surveillance systems. These technological solutions have been reported to improve timeliness and the speed at which epidemiological data analysis is performed, which will facilitate rapid response. Malaria cases reported through the paper-based system took more than 24-h, the reason for this is that paper notification forms rely on the case investigators to fetch the reports from the healthcare facilities, as the healthcare facilities have no faxing facilities. Even though these cases will be investigated upon being picked up, there are delays in entering the information into a centralized database, so that relevant stakeholders can have access to it for decision-making. In addition, it also delays detection of any epidemics.

There were some outliers in the timeliness of reporting of cases with the 24-h reporting, this was evident in instances where health care facilities had an over flow in patients, which resulted in the increased burden for the healthcare facility worker diagnosing, treating and notifying malaria. It was noted in some instances a notification, either a paper-based, a 24-h reporting notification or both was only completed at the end of the healthcare worker’s shift, at the end of the week or at the end of the month. This is a major challenge in the country and this is not only with the notification of malaria cases, but other NMC.

The findings on timeliness supports several studies [6, 8] A study by Blumberg et al., who implemented a technical framework for 8 months, where a nurse was hired to use a mobile phone to report malaria cases to the provincial malaria control programmes through short message service (SMS) notification [8]. The study found that the SMS notification for each diagnosed malaria case improved the timeliness of data transmission [8]. Of the 23 cases, 18 had basic information entered into the provincial malaria information system within 24-h [8].

In a study by Huaman et al. 40 sites, of which 18 were clinics and 22 were ships were included in a 12 week prospective evaluation, which aimed at assessing the timeliness and data quality of the cases reported [6]. The sites were divided into three groups (phone group—those who received phones and were expected to report through the phones, visit group—those who were visited once a month, but continued their normal reporting procedure and the control group—those who did not receive any interventions and continued with their normal reporting procedure) [6]. What they found was that only the timeliness of those in the phone group had improved (from 64.4 to 84%) in the clinics and from 46.9 to 77.3% on ships [6].

The healthcare workers found the 24-h mobile reporting system simpler to use than the paper-based system and this contradicts the study findings on data validity. A significant number of healthcare workers could finish filling in the report within 2 min or between 2 and 5 min reporting a case through the 24-h mobile reporting system. This could be the reason why the healthcare workers perceived the system to be easier to use. Separate studies have shown that mobile technology is an innovative way to decrease the reporting load by up to 50% [11]. Given these results, this study suggests that the 24-h mobile reporting system can be an innovative way to improve timeliness of case reporting from facilities in South Africa and phase out the cumbersome paper-based reporting system [11].

Although the acceptability was not 100%, most of the health care workers were willing to continue using the system. The reason for this could be that the paper-based system notification form consists of unstructured or less structured free form texts, while the 24-h mobile reporting is standardized and has fewer free form texts, which allows for easier completion. The healthcare workers that found the mobile tool to be challenging were mostly healthcare workers, who are nearing pension. This is mostly, because they found it hard to operate cell phone devices, even in instances were the healthcare workers was not using a smart device. There were two types of groups during training, those that have technological assets and have an optimistic view towards electronic platforms and those that are disconnected from electronic tools, both psychologically and physically.

The use of the 24-h mobile reporting could be highly acceptable because of its cost effectiveness compared to the paper-based system. The USSD is a technology that allows users who do not have ‘data bundles’ loaded on their handsets, to nevertheless interact with data-type services. The ubiquity of USSD in Africa, its low cost and its familiarity to end users are key advantages.

There are technical challenges associated with mobile technology. One of these challenges is network problems. The location of the healthcare facilities is in remote areas, where network signal still remains a problem. Developing countries have limited infrastructure to prevent failure to connect to a network. In a survey conducted by the Independent Communications Department of South Africa, 95% of respondents said their cellular network service level had declined in the past 3 months (July 2016–September 2016) [15].

## Limitations and recommendations

The questionnaire was administered during the malaria season but the numbers of cases were lower than expected due to the strides the South African Malaria programme has made in decreasing malaria incidence in the country. Private healthcare facilities were excluded from the study, which might have an impact on the findings of the study.

Due to the low number of cases there were also a few healthcare workers that used the 24-h mobile reporting system and because of healthcare worker turnover most of the healthcare workers that reported cases through the 24-h mobile reporting system were not present at the time of questionnaire administration.

The timeliness in data input was better using the 24-h mobile reporting system, compared to the paper-based system, but the expected response might be compromised due to the wide range in the median for the 24-h mobile reporting tool.

There might have been recall bias of the time it took for healthcare workers to fill in both the 24-h mobile reporting system electronic forms and the paper-based system notification form; and this is particularly true for cross-sectional surveys.

There are no daily platforms to remind healthcare workers to report cases. Reporting delays have been shown to be because of forgetfulness to report cases. Healthcare workers in public healthcare facilities are overloaded with patients and paperwork during and/or after consulting a patient and disease reporting is not considered a priority. This is especially true in developing countries, where a healthcare worker attends to an average of > 30 patients a day. A study by Huaman et al. showed that contacting the patient a few hours before the reports should be in improved reporting rates and the timeliness of the reports [6]. It is not feasible for the malaria programmes to call every healthcare worker that notifies malaria cases, at every healthcare facility across all malaria endemic districts; more feasible tools, such as automated phone calls (calls placed without any human input other than the recipient) and messaging will need to be explored in order to improve both the reporting rates and the timeliness of the reports [6].

The use of the 24-h mobile reporting system is limited to healthcare workers although laboratory technicians should also be able to use it. Its use should also be scaled up to other provinces in order to standardize reporting practices, not only for malaria, but for other NMC.

## Conclusions

Previous studies have shown that mobile technology can reduce the time required for cases to be reported from the healthcare facility to the malaria programme. The WHO proposes that all countries striving to eliminate malaria by 2018 should report malaria within 24-h, investigate the cases within 48 h and place targeted interventions within 72 h. Thus far the 24-h mobile reporting System has been effective at this, regardless of the network problems that might be experienced by the healthcare workers.

## Abbreviations

MIS: malaria information system
NDOH: National Department of Health
NICD: National Institute of Communicable Disease
NMC: notifiable medical conditions
SHSPH: School of Health Systems and Public Health
SMS: short message service
USSD: Unstructured Supplementary Service Data
WHO: World Health Organization
